# A general equilibrium approach to pricing volatility risk

**DOI:** 10.1371/journal.pone.0215032

**Published:** 2019-04-12

**Authors:** Jianlei Han, Martina Linnenluecke, Zhangxin Liu, Zheyao Pan, Tom Smith

**Affiliations:** 1 Department of Applied Finance, Macquarie University, NSW, Australia; 2 UWA Business School, The University of Western Australia, Crawley, WA, Australia; Institute for Economic Forecasting, Romanian Academy, ROMANIA

## Abstract

This paper provides a general equilibrium approach to pricing volatility. Existing models (e.g., ARCH/GARCH, stochastic volatility) take a statistical approach to estimating volatility, volatility indices (e.g., CBOE VIX) use a weighted combination of options, and utility based models assume a specific type of preferences. In contrast we treat volatility as an asset and price it using the general equilibrium state pricing framework. Our results show that the general equilibrium volatility method developed in this paper provides superior forecasting ability for realized volatility and serves as an effective fear gauge. We demonstrate the flexibility and generality of our approach by pricing downside risk and upside opportunity. Finally, we show that the superior forecasting ability of our approach generates significant economic value through volatility timing.

## Introduction

Volatility modelling has proceeded as a field separate from asset pricing. Statistical models, such as ARCH [1, 2], GARCH [3], stochastic volatility [4], and option prices [5] are commonly used to estimate volatility, without reference to modern asset pricing theory. In this paper, we propose to *price* volatility using a general equilibrium asset-pricing framework. The advantage of such an approach is that volatility can be priced and measured in the most general setting available. The approach also allows us to extend measurement and pricing (such as downside risk pricing and upside opportunity pricing), which cannot be achieved with current approaches to volatility modelling.

This paper assumes a complete market setting where state prices are available for each time and state. State prices are derived from the general equilibrium state pricing framework [6]. Options complete the state space [7] and in this compete market all investors face the same state prices and these prices can be used to price any asset in the aggregated market. State prices are obtained for each time based on options written on the aggregate market (S&P 500 index as a proxy)[8]. We apply this state pricing approach to market volatility risk and are able to derive prices that are almost perfectly correlated with the CBOE Volatility Index (VIX) but are the result of a general equilibrium model. There are several advantages to treating volatility as any other asset and pricing it using the general equilibrium approach [6]. First, we treat volatility pricing the same as any other pricing exercise. Second, we do not have to assume a specific form of utility preferences (i.e., mean-variance or mean-variance-skewness or any other form) to implement this approach. Third, the equilibrium price of volatility provides not only a more general approach that is very flexible and can also be used for individual securities, but also serves as a better predictor of future volatility and as an investor fear gauge.

Empirically, we generate market state prices from the S&P 500 index options and use them to price the 49 Fama and French industry portfolio’s volatility. Although our method can be applied to any traded asset, we focus on industries since understanding the volatility of a particular industry is crucial for both investors and policy makers [9, 10]. The ex-ante industry volatility measure constructed in this approach yields better forecasts of realized volatility than existing approaches and serves as a qualified investor fear gauge. We then demonstrate the flexibility and generality of our approach by pricing downside and upside opportunity, which complements the prior work [11–14]. We show that these new upside and downside volatility measures work well empirically. Finally, we analyze the economic value of volatility timing using the general equilibrium measures versus existing volatility measures. We show that the superior forecasting ability of our general equilibrium volatility measure has greater economic value for investors wishing to manage volatility.

Our paper is related to the recent literature on understanding volatility in a general equilibrium framework. As an extension of [15] and [16], Tauchen [17] proposes a consumption-based general equilibrium model that assumes stochastic consumption. The model generates a two-factor structure for stock market volatility along with time-varying risk premiums on consumptions and volatility risk, and the leverage effect. In the general equilibrium framework, Bansal *et al*.[18] demonstrate that besides the cash flow risk and discount rate risk, volatility risk is an important and separate risk source that cannot be ignored. In contrast to this strand of literature, we impose no assumptions on consumption dynamics and rely only on state prices extracted from the options market. The focus of these existing studies is to explain the stylized facts (e.g., leverage effect, equity risk premium) in the market, while we aim to provide market participants with an easy and flexible tool to measure and manage the volatilities of asset portfolios or individual securities.

Our paper is also related to the prior literature on constructing volatility indices based on options. As long established in the literature, ex-ante risk-neutral volatility can be built upon the fair value of future variance. Britten-Jones and Neuberger [19] use a replicating strategy to synthesize a variance swap using options contracts, assuming continuity in the underlying asset price. Jiang and Tian [20] build on a similar concept by incorporating a jump-diffusion stochastic volatility model. More recently, Martin [21] proposes a market level volatility index as the price of squared returns contract and proves it serves as the lower bound for the market risk premium. Martin and Wagner [22] extend the method of Martin [21] and develop a stock level volatility index and link it with the expected stock return. Sharing the same spirit of Martin [21] and the other existing literature, we also treat volatility (or more precisely, the squared return) as an asset. However, we adopt the general equilibrium approach and are able to construct volatility indices for assets without the need to use traded options. In contrast, Martin and Wagner [22]’s methodology can only be applied to individual stocks with the availability of options. Since options either do not exist or are illiquid for most stocks/industry portfolios, our approach is more general and has a wider application.

Our paper also adds to recent efforts to disentangle the effects of upside and downside uncertainty on asset prices [11, 14] by proposing a new approach for volatility decomposition. One common approach for decomposing volatility into upside and downside components is to use a threshold to compute risk-neutral expectations of semi-variances [12, 13, 23]. However, this approach still depends on the existence of traded options, while our approach does not.

The paper is organized as follows. Section 2 outlines volatility pricing using a general equilibrium model. Section 3 extends our approach to price downside risk and upside opportunity. Section 4 analyzes the economic value of our volatility measures in a volatility timing framework. Section 5 concludes.

## Methods and materials

### Pricing volatility in a general equilibrium model

This section outlines the general equilibrium approach for pricing market volatility and shows how it can be extended to pricing industry volatility. Industry volatility prices are compared with existing approaches to forecasting realized volatility and evaluated as a gauge of investor fear. We have obtained the appropriate permissions for use of third-party data and complied with the terms of service for the websites from which we collected data.

Under a state pricing approach, the value of any asset is the sum of the state prices multiplied by the payoff in each state. If, for example, we were to price the market portfolio *M* which pays off *F*_*ms*_ in each of *S* states one period (set as 30 days in this paper) from now, the price is given by:

$$P_{m}=\sum_{s=1}^{S}\phi_{ms}F_{ms}$$ (1)

Breeden and Litzenberger [8] argue that the market portfolio, as a proxy for aggregate consumption, is sufficient to represent the different states in the economy. We show in S1 Appendix how we obtain the state prices using market options, where the market is represented by S&P 500 index (SPX).

For an arbitrage asset *i*, whose payoff *F*_*i*_ depends on the level of the market, under the complete market setting, its price is given by:

$$P_{i}=\sum_{s=1}^{S}\phi_{ms}E[F_{is}|F_{ms}]$$ (2)

If we were to take a linear projection of *F*_*i*_ onto *F*_*M*_, then we would obtain:

$$P_{i}=\sum_{s=1}^{S}\phi_{ms}[\alpha_{i}+\beta_{i}F_{ms}]$$ (3)

or

$$P_{i}=\alpha_{rfi}+\beta_{i}P_{m}$$ (4)

Since $\sum_{s=1}^{S}\phi_{ms}$ is the price of a risk-free asset with payoff of 1, *α*_*rfi*_ is the price of a riskless asset with payoff *α*_*i*_.

This is a relation that closely resembles the Sharpe-Lintner Capital Asset Pricing Model [24]; however, the derivation contains obvious differences. First, the market price of risk will vary over time as the state prices change. Second, the risk-free factor will be different for each asset *i* depending on the magnitude of *α*_*i*_. Moreover, a nonlinear projection of the conditional expectation leads to the mean-variance-skewness model of Kraus and Litzenberger [25]. S2: Appendix provides a more detailed discussion of the relation between state pricing theory, the CAPM and the co-skewness pricing.

We now consider pricing market volatility. Here, the payoff is the squared market return at each state. The price of market volatility under the general equilibrium state-pricing approach is given by:

$$SVX_{M}^{2}=\sum_{s=1}^{S}\phi_{ms}R_{ms}^{2}$$ (5)

where *SVX*_*M*_ is the state pricing volatility index for the market. It is the general equilibrium price of market volatility.

Compared to the calculation of the VIX, the *SVX*_*M*_ formula offers a more straightforward approach. For a more detailed discussion on how to construct *SVX*_*M*_ and how it performs against other volatility measures in predicting future market volatility, see [26]. The approach of using Arrow-Debreu securities to price squared returns has also been adopted in prior studies, see [27–29]. To price volatility on an arbitrary asset, for example industry *I*, the approach above yields:

$$SVX_{I}^{2}=\sum_{s=1}^{S}\phi_{ms}E[R_{Is}^{2}|R_{ms}^{2}]$$ (6)

An assumption of a linear relation between individual asset return and market return (as in Eq 3) would lead to a linear relation between individual asset return squared and market return squared conditional on the given market return, *R*_*m*_. Naturally, we have:

$$SVX_{I}^{2}=\sum_{s=1}^{S}\phi_{ms}[\alpha_{I}+\beta_{I}R_{ms}^{2}]$$ (7)

or

$$SVX_{I}^{2}=\alpha_{rfI}+\beta_{I}SVX_{M}^{2}$$ (8)

where *α*_*rfI*_ is the price of a riskless asset with payoff *α*_*I*_, $SVX_{M}^{2}$ is as defined above in Eq 5.

The details of the construction of *SVX*_*I*_ are presented in S3: Appendix. We see that under the linear projection approach, the volatility price of any asset depends on the market price of volatility in a straightforward manner.

We compare two other volatility measures to our measure. The first is an ad-hoc industry volatility index using the widely available CBOE volatility index VIX. To achieve that, we simply replace $SVX_{M}^{2}$ by $VIX_{M}^{2}$:

$$VIX_{I}^{2}=\alpha_{rfI}+\beta_{I}VIX_{M}^{2}$$ (9)

where *VIX*_*M*_ is the CBOE VIX.

The second measure is the historical volatility, *HV*_*I*_, which is the realized volatility in the previous year.

### Data set

To estimate state prices in the complete market setting, we obtain prices and implied volatilities of S&P 500 index options and S&P 500 index dividend yields from the Ivy DB US Option Metrics, available through Wharton Research Data Services. The options data are available on a daily basis from January 4, 1996 to April 29, 2016. Interest rates are taken from the Center for Research in Security Prices (CRSP) Zero Curve file. We apply a cubic spline to the interest rate term-structure data to match the length of the risk-free rate with the corresponding option maturity. The 49 industry portfolios are obtained from Kenneth R. French’s Data Library (http://mba.tuck.dartmouth.edu/pages/faculty/ken.french/data_library.html) and has been provided in S1 Data. Given we need to estimate the betas in Eq 7 using a fixed two-year rolling window, we examine daily industry returns since January 1994.

## Results and discussions

### Summary statistics of industry volatility indices

Summary statistics of *SVX*_*I*_ are reported in Table 1. The mean of the annualized *SVX*_*I*_ varies across industries, ranging from 0.129 for the utility industry to 0.273 for the coal industry. Consistent with economic intuition, the “necessities” industries, such as Food, Soda, Beer, Smoke, and Utilities [30] are insensitive to business cycles, and are the least volatile, on average.

**Table 1:** Summary statistics of industry SVX_I_.

Industry	Obs	Mean	Std. Dev.	Min	Max
Agric	5,099	0.150	0.103	0.039	0.950
Food	5,099	0.132	0.051	0.052	0.482
Soda	5,099	0.138	0.062	0.005	0.602
Beer	5,099	0.136	0.067	0.044	0.481
Smoke	5,099	0.143	0.066	0.049	0.645
Toys	5,099	0.180	0.066	0.080	0.603
Fun	5,099	0.216	0.098	0.088	0.893
Books	5,099	0.165	0.079	0.060	0.772
Hshld	5,099	0.145	0.060	0.059	0.534
Clths	5,099	0.179	0.078	0.066	0.714
Hlth	5,099	0.155	0.066	0.055	0.553
MedEq	5,099	0.160	0.063	0.059	0.581
Drugs	5,099	0.164	0.065	0.068	0.558
Chems	5,099	0.188	0.083	0.091	0.818
Rubbr	5,099	0.155	0.061	0.083	0.557
Txtls	5,099	0.166	0.076	0.066	0.711
BldMt	5,099	0.185	0.072	0.080	0.686
Cnstr	5,099	0.224	0.095	0.097	1.080
Steel	5,099	0.243	0.126	0.084	1.207
FabPr	5,099	0.182	0.086	0.056	0.780
Mach	5,099	0.210	0.088	0.104	0.863
ElcEq	5,099	0.211	0.081	0.105	0.798
Autos	5,099	0.212	0.089	0.101	0.839
Aero	5,099	0.203	0.098	0.089	0.756
Ships	5,099	0.168	0.073	0.073	0.784
Guns	5,099	0.146	0.067	0.028	0.554
Gold	5,043	0.184	0.102	0.005	0.925
Mines	5,099	0.208	0.133	0.049	1.199
Coal	4,735	0.273	0.154	0.007	1.363
Oil	5,099	0.182	0.106	0.039	1.068
Util	5,099	0.129	0.076	0.045	0.735
Telcm	5,099	0.184	0.083	0.075	0.836
PerSv	5,099	0.171	0.063	0.079	0.601
BusSv	5,099	0.177	0.066	0.084	0.615
Hardw	5,099	0.250	0.134	0.093	1.009
Softw	5,099	0.224	0.105	0.090	0.770
Chips	5,099	0.244	0.119	0.089	0.893
LabEq	5,099	0.204	0.081	0.095	0.667
Paper	5,099	0.162	0.061	0.085	0.631
Boxes	5,099	0.171	0.069	0.072	0.678
Trans	5,099	0.181	0.069	0.088	0.619
Whlsl	5,099	0.156	0.059	0.078	0.605
Rtail	5,099	0.182	0.074	0.073	0.626
Meals	5,099	0.156	0.056	0.070	0.549
Banks	5,099	0.229	0.114	0.083	1.494
Insur	5,099	0.191	0.096	0.077	1.005
RlEst	5,099	0.164	0.119	0.033	0.950
Fin	5,099	0.263	0.132	0.094	1.331
Other	5,099	0.179	0.088	0.060	0.669

This table presents summary statistics of Industry SVX_I_ of 49 industry portfolios. The data are from 4 January 1996 to 29 April 2016.

**Fig 1:**
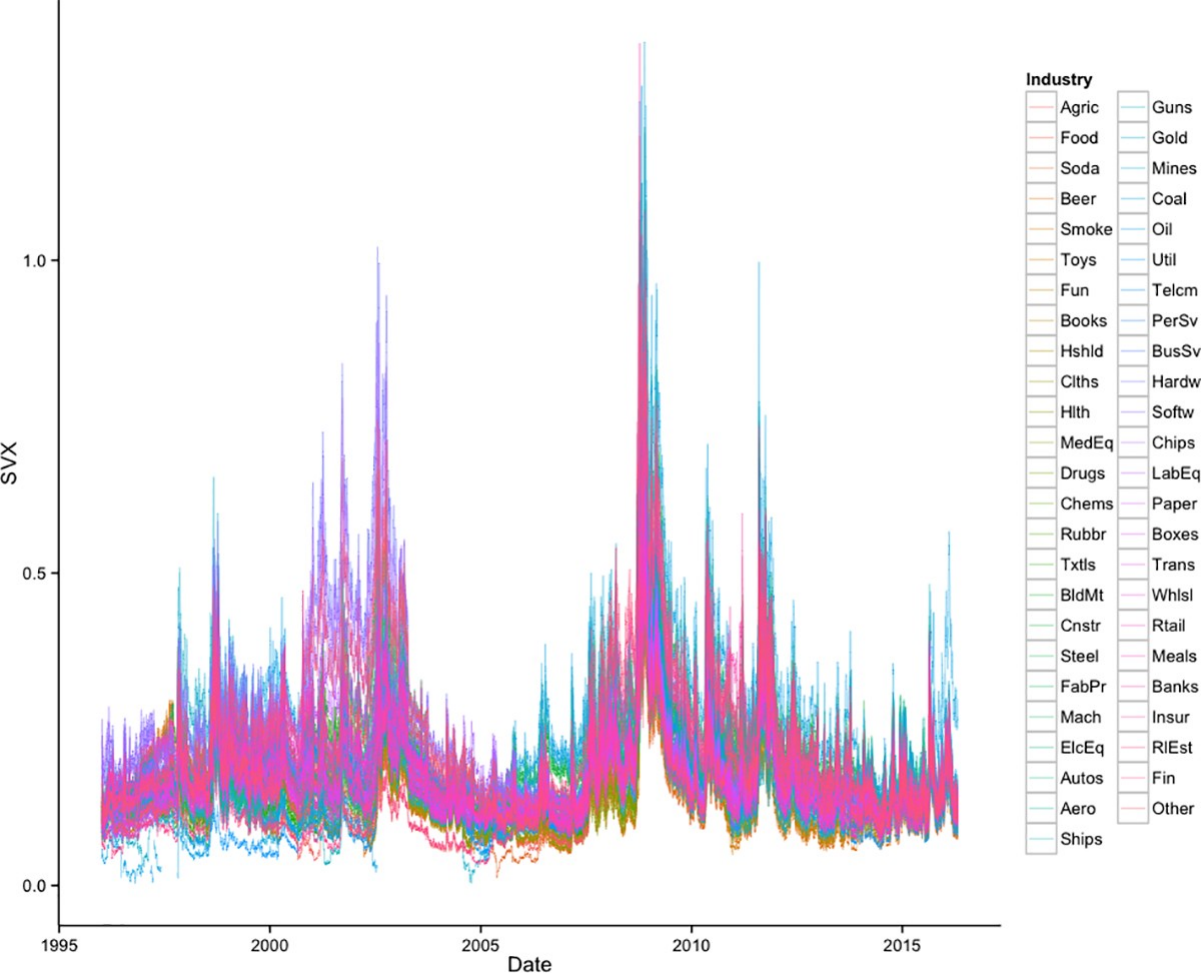
SVX_I_ for 49 industries: 1996–2016. This figure plots the Industry SVX_I_ of 49 industry portfolios. The data are from 4 January 1996 to 29 April 2016.

*SVX*_*I*_ for the 49 industry portfolios is illustrated in Fig 1. It is apparent that there is a strong positive correlation among the volatility indices. Fig 1 also reveals that there is a significant time variation in volatility for all industries in the analysis, showing an upward spike in the 1998 Asian Financial Crisis and during the technology bubble of the early 2000’s. A peak occurs around the 2008 Global Financial Crisis.

### Forecasting future 30-day realized volatility

The primary goal of a volatility index is to serve as a measure of the next 30-day expected volatility [31]. In this section, we examine the information content of *SVX*_*I*_ in predicting subsequent 30-day realized return volatility from January 1996 to April 2016. We regress the future 30-day close-to-close realized volatility on different volatility measures in the following models:

$$RV_{I,t,t+30}=\alpha_{I}+\beta_{I}SVX_{I,t}+\varepsilon_{I,t,t+30}$$ (10)

where *I* stands for one of the 49 industry portfolios, *RV*_*I*,*t*,*t*+30_ denotes the annualized realized volatility over the next 30 days and it is defined as $RV_{I,t,t+30}=100(\frac{365}{30}\sum_{i=1}^{22}R_{i}^{2}{)}^{1/2}$, where $R_{i}^{2}$ is the square of the daily portfolio return. We also run this regression for *VIX*_*I*_ and *HV*_*I*_, where *HV*_*I*_ is the annualized realized volatility in the previous year. These measures are highly correlated on average, so we cannot include both in the same regression to determine which index is more statistically significant. Instead, we use the Mincer and Zarnowitz [32] regression to compare the prediction performances of these three volatility measures.

To be an unbiased volatility predictor, we expect alphas to be not significantly different from 0 and betas to be not significantly different from 1. If *SVX*_*I*_ is a better predictor than the other measures, the forecasting regression using it as the predictor is expected to generate the highest model explanatory power, as expressed in adjusted R-squared.

We run the above regressions for each of 49 industry portfolios and report the arithmetic average of regression results in Table 2. The covariance matrix is computed according to Newey and West [33] to correct for any potential serial correlations in the error terms.

**Table 2:** Forecasting realized volatility with SVX_I,_ VIX_I,_ and HV_I_.

Dep Variable: RV_I_	Average of R2	Average of Intercept	Average of *β* for SVX_I_	Average of *β* for VIX_I_	Average of *β* for HV_I_
	47.2%				
Coef		0.046**	1.010***		
p-value		0.041	1.15E-133		
Std Err		0.003	0.015		
	45.8%				
Coef		0.044*		0.918***	
p-value		0.057		3.6E-100	
Std Err		0.003		0.014	
	28.2%				
Coef		0.072***			0.677***
p-value		1.3E-25			9.2E-137
Std Err		0.004			0.015

This table presents the average OLS estimates of regressions in Eq (10). The regressions take the following general form

Y=α+βX+ε

All coefficients, p-values, and standard errors are an average of the corresponding measures from 49 regressions on all industry portfolios. Dependent variable RVI is annualized realized volatility in future 30 days of one of 49 industry portfolios, where $RV=100\times (365/30\times \sum_{i=1}^{22}R_{i}^{2}{)}^{1/2}$ and R_i_ is the daily portfolio return. The data are from 4 January 1996 to 29 April 2016. To correct for autocorrelation and heteroskedasiticity, we use the Newey-West estimator for covariance matric with automatically selected lags as in Newey and West [33].

***, **, and * denote significance at the 0.01, 0.05, and 0.10 level, respectively.

The mean level of the *β* of *SVX*_*I*_ is 1.01, with an average standard error of 1.5%. As expected, *β* of *SVX*_*I*_ is significantly different from 0 at 1% level, and not significantly different from 1 at any levels. In comparison, *β* of *VIX*_*I*_ has a mean level of 0.918 with a standard error of 1.4%. It is significantly different from both 0 and 1 at 1% level. *β* of *HV*_*I*_ has the lowest mean level out of the three measures; namely, 0.677 with a standard error of 1.5%. On average, *α* of *SVX*_*I*_ is 0.046, which is marginally significantly different from 0 at 5% level. The mean for α of *VIX*_*I*_ is 0.04 and is not significantly different from 0 at 5% level. In comparison, the α of *HV*_*I*_ has an average value of 0.072 and is significantly different from 0. *SVX*_*I*_ provides higher adjusted R-squared than the regressions using *VIX*_*I*_ and *HV*_*I*_. On average, the model with *SVX*_*I*_ as the predictor has a 1.4% higher adjusted R-squared than *VIX*_*I*_ and 19% higher than *HV*_*I*_. Therefore, we conclude that the state price volatility index *SVX*_*I*_ is a more efficient forecaster of future realized volatility than its counterparts. This industry-level result reinforces Liu and O'Neill [26]’s finding that state price volatility outperforms *VIX* and other predictors at the market level.

### Fear gauge

It is well-documented that there is a negative correlation between the rate of change in volatility (e.g., CBOE VIX) and daily market returns [34]. As expected, market volatility increases, investors demand a higher rate of return on stocks and prices fall, which ultimately leads to a drop in the current market return. Therefore, we study the contemporaneous relation between rates of change in various industry volatility measures and daily industry portfolio returns. In particular, we investigate whether these indices contain any fear information from the market state prices. Generally, a fall in an industry portfolio usually implies a rally in investor fear in the segment. Therefore, we expect to see negative betas in all volatility measures. A fear gauge, such as CBOE VIX [35], should respond more to negative changes in portfolio returns than positive changes. We are interested in testing whether industry state price volatility measures can capture this asymmetric fear gauge effect. We regress the daily changes of various measures against industry portfolio returns in the following forms:

$$\Delta SVX_{I,t}=\alpha_{I}+\beta_{1,I}R_{I,t}+\beta_{2,I}R_{I,t}^{-}+\varepsilon_{I,t}$$ (11)

where *I* stands for each of the 49 industry portfolios, Δ measures the daily changes, and *R*_*I*,*t*_ is the daily industry portfolio return, $R_{I,t}^{-}$ is defined as min(*R*_*I*,*t*_,0).

We run the above regressions for each of 49 industry portfolios and report the arithmetic average of regression results in Table 3. The covariance matrix is computed according to Newey and West [33] to correct for any potential serial correlations in the error terms. On average, *β*_1_ is significantly less than 0 at the 1% level, implying that there is an inverse relation between contemporaneous changes in volatility indices and changes in portfolio returns. *β*_2_ is also significantly less than 0 at the 1% level. These results show that the response to different swings in portfolio returns is strongly asymmetric, and are consistent with the findings of [35] and [26]. Besides statistical significance, the coefficients are also economically meaningful: on average, an increase in the industry return by 100 basis would result in 51.7 basis decrease in *SVX*_*I*_ and an decrease in the industry return by 100 basis would lead to 73.8 basis increase in *SVX*_*I*_.

**Table 3:** Regression results of rate change of SVX_I_ against returns of industry portfolios.

Dep Variable: ΔSVX_I_	Average of R2	Average of Intercept	Average of *β*_1_ for R_I,t_	Average of *β*_2_ for R_I,t_^-^
	36.7%			
Coef		-0.001*	-0.517***	-0.221***
p-value		0.051	0.000	0.001
Std Err		0.000	0.019	0.032

This table presents the average OLS estimates of regressions in Eq (11). The regressions take the following general form

$\Delta SVX_{I,t}=\alpha +\beta_{1}R_{I,t}+\beta_{2}R_{I,t}^{-}+\varepsilon$

All coefficients, p-values, and standard errors are an average of the corresponding measures from 49 regressions on all industry portfolios. Independent variables include RI, daily return of the corresponding industry portfolio; and RI, daily return of the corresponding industry portfolio conditional on whether the return is below 0, i.e., RI^-^ = min(RI, 0). The dependent variable is the daily return of SVXI, where ΔSVX_I,t_ = ln(SVX_I,t_/SVX_I,t−1_). The return data are from 4 January 1996 to 29 April 2016. To correct for autocorrelation and heteroskedasiticity, we use the Newey-West estimator for covariance matric with automatically selected lags as in Newey and West [33].

***, **, and * denote significance at the 0.01, 0.05, and 0.10 level, respectively.

### Downside risk and upside opportunity in a general equilibrium model

Recent literature highlights the importance of distinguishing downside and upside volatility risk [11, 14, 36]. Here, we use the general pricing approach to price downside risk and upside risk.

Downside market risk is given by:

$$BEX_{M}=\sqrt{\sum_{s=1}^{S}\phi_{ms}R_{ms}^{2}I_{R_{ms}<0}}$$ (12)

where $I_{R_{ms}<0}$ is an indicator variable equal to 1 if *R*_*ms*_ is less than zero.

Similarly, upside market risk is given by:

$$BUX_{M}=\sqrt{\sum_{s=1}^{S}\phi_{ms}R_{ms}^{2}I_{R_{ms}>0}}$$ (13)

where $I_{R_{ms}>0}$ is an indicator variable equal to 1 if *R*_*ms*_ is greater than zero.

Industry measures of *BEX* and *BUX* are obtained in an analogous manner to industry volatility *SVX*_*I*_ (Eq 7) and can be represented as:

$$BEX_{I}^{2}=\alpha_{Down,I}+\beta_{Down,I}BEX_{M}^{2}$$ (14)

$$BUX_{I}^{2}=\alpha_{Up,I}+\beta_{Up,I}BUX_{M}^{2}$$ (15)

To estimate the alphas and betas in Eq 14 and Eq 15, we use a linear least squares regression of squared daily industry returns on squared S&P 500 market returns. Specifically, we are interested in the alphas and betas in the following regressions:

$$R_{I}^{2}=\alpha_{Down,I}+\beta_{Down,I}R_{M|Down}^{2}+\varepsilon_{I}$$ (16)

$$R_{I}^{2}=\alpha_{Up,I}+\beta_{Up,I}R_{M|Up}^{2}+\varepsilon_{I}$$ (17)

where $R_{I}^{2}$ is the daily squared industry return, and $R_{M|Down}^{2}$ ($R_{M|Up}^{2}$) is the market return squared conditional on whether the market has gone down (up) from the previous day, regardless of movement in the industry portfolio. We also considered different definitions of conditional downturn return squared for the industry portfolio. We selected the current definition because it is more meaningful for examining how the industry portfolio responds and contributes to a downturn in the whole market. The return is computed using the closing value at the end of day. We estimate each beta using a two-year fixed rolling window. That is, on the 505^th^ day, we use the past two years (504 trading days) of return squared to estimate the alphas and betas in the above regressions.

### Industry state-price volatility that incorporates downside market volatility

Prior studies have shown that returns become more correlated in a bear market (e.g., [37, 38]). As a result, we extend the basic formula for *SVX*_*I*_ by using an alternative linear projection that incorporates market downside movement:

$${\left(SVX_{I}^{D}\right)}^{2}=\sum_{s=1}^{S}\phi_{ms}[\alpha_{I}+\beta_{I}R_{ms}^{2}+\gamma_{I}R_{ms}^{2}I_{R_{ms}\leq 0}]$$ (18)

Which can be solved to yield:

$${\left(SVX_{I}^{D}\right)}^{2}=\alpha_{rfI}+\beta_{I}SVX_{M}^{2}+\gamma_{I}BEX_{M}^{2}$$ (19)

where *α*_*rfI*_ is the price of a riskless asset with payoff *α*_*I*_, $SVX_{M}^{2}$ is as defined above in Eq 4. and $I_{R_{ms}\leq 0}$ is an indicator variable equal to 1 if *R*_*ms*_ is less than zero. In this definition, the volatility price of any asset depends on its sensitivity to the price of market volatility and (in addition) to the price of market downside volatility.

To estimate the alphas, betas, and gammas in Eq 19, we use a linear least square regression of squared daily industry returns on squared daily S&P 500 market returns.

$$R_{I}^{2}=\alpha_{Down,I}+\beta_{Down,I}R_{M}^{2}+\gamma_{Down,I}R_{M|Down}^{2}+\varepsilon_{I}$$ (20)

where $R_{I}^{2}$ is the daily squared industry return, and $R_{M|Down}^{2}$ is the market return squared conditional on whether the market has gone down from the previous day, regardless of movement in the industry portfolio.

### Forecasting future 30-day realized volatility with $SVX_{I}^{D}$

To extend the analysis described in Section 2.3, we examine the information content of $SVX_{I}^{D}$ in predicting the future 30-day realized volatility in each industry portfolio. We regress the future 30-day close-to-close realized volatility on different volatility measures in the following models:

$$RV_{I,t,t+30}=\alpha_{I}+\beta_{I}SVX_{I,t}^{D}+\varepsilon_{I,t,t+30}$$ (21)

Results are reported in Table 4 Panel A. The mean level of β of $SVX_{I}^{D}$ is 1.003, with an average standard error of 1.5%. β of $SVX_{I}^{D}$ is significantly different from 0 at 1% level, and not significantly different from 1 at any levels. Comparing to results in Table 2, we can see that $SVX_{I}^{D}$ outperforms other volatility candidates in terms of adjusted R-squared.

**Table 4:** Forecasting realized volatility and downside realized volatility with SVX^D^_I_ and BEX_I_.

	Dep Variable	Average of R2	Average of Intercept	Average of *β* for SVX^D^_I_	Average of *β* for BEX_I_
**Panel A**	**RV**_**I**_	47.3%			
Coef			0.047**	1.003***	
p-value			0.046	1.27E-133	
Std Err			0.003	0.015	
**Panel B**	**RV**^**D**^_**I**_	34.7%			
Coef			0.029*		0.917***
p-value			0.058		8.3E-166
Std Err			0.003		0.018

This table presents the average OLS estimates of regressions in Sections III C and D. The regressions take the following general form

Y=α+βX+ε

All coefficients, p-values, and standard errors are an average of the corresponding measures from 49 regressions on all industry portfolios. Dependent variables include; (1) RV is annualized realized volatility in future 30 days of one of 49 industry portfolios, where $RV=100\times (365/30\times \sum_{i=1}^{22}R_{i}^{2}{)}^{1/2}$ and R_i_ is the daily portfolio return; and (2) RVDI is the realized downside volatility in future 30 days of one of 49 industry portfolios, where $RVD=100\times (365/30\times \sum_{i=1}^{22}R_{i}^{2}I_{R_{i}\leq 0}{)}^{1/2}$. The data are from 4 January 1996 to 29 April 2016. To correct for autocorrelation and heteroskedasiticity, we use the Newey-West estimator for covariance matric with automatically selected lags as in Newey and West [33].

***, **, and * denote significance at the 0.01, 0.05, and 0.10 level, respectively.

### Forecasting future 30-day realized downside volatility

A typical volatility measure does not describe the proportion of upside gain versus downside threat. In this paper, we solve this problem by introducing a downside (upside) volatility index *BEX*_*I*_(*BUX*_*I*_) for each industry portfolio as an *ex-ante* predictor of future downside (upside) volatility. It is important to note that, unlike the comparison with *SVX*_*I*_ and *VIX*_*I*_ in the previous section, we do not have a VIX benchmark per se because is not mathematically feasible to derive a downside VIX using market state prices. We regress the future 30-day close-to-close realized downside volatility in the following way:

$$RVD_{I,t,t+30}=\alpha_{I}+\beta_{I}BEX_{I,t}^{2}+\varepsilon_{I,t,t+30}$$ (22)

where *I* stands for each of the 49 industry portfolios, *RVD*_*I*,*t*,*t*+30_ denotes the realized downside volatility over the next 30 days and it is defined as $RVD_{I,t,t+30}=100(\frac{365}{30}\sum_{i=1}^{22}R_{i=1}^{22}I_{R_{i}\leq 0}{)}^{1/2}$.

We expect alphas to be not significantly different from 0 and betas to be not significantly different from 1 if *BEX*_*I*_ is an unbiased forecaster, and betas to be significantly different from 0. We run the above regression for each of 49 industry portfolios and report the arithmetic average of regression results in Panel B in Table 4. The covariance matrix is computed according to Newey and West [33] to correct for any potential serial correlations in the error terms. The mean level of *β* of *BEX*_*I*_ is 0.917, with an average standard error of 1.8%. *β* of *BEX*_*I*_ is significantly different from 0 and 1 at 1% level. On average, *α* of *BEX*_*I*_ is 0.029 and is not significantly different from 0 at 5% level. The average adjusted R-squared of Eq 22 is 34.7%. We show that *BEX*_*I*_ is an efficient forecaster of future realized downside volatility.

### Fear gauge property of $SVX_{I}^{D}$

We further study the contemporaneous relation between rates of change in $SVX_{I}^{D}$ and daily industry portfolio returns. We are interested in testing whether the modified industry volatility measure can better capture the fear gauge. We regress the daily changes of $SVX_{I}^{D}$ against the industry portfolio returns in the following forms:

$$\Delta SVX_{I,t}^{D}=\alpha_{I}+\beta_{1,I}R_{I,t}+\beta_{2,I}R_{I,t}^{-}+\varepsilon_{I,t}$$ (23)

where *I* stands for each of the 49 industry portfolios, Δ measures the daily changes, and *R*_*I*,*t*_ is the daily industry portfolio return, $R_{I,t}^{-}$ is defined as min(*R*_*I*,*t*_,0). We perform a similar analysis for $\Delta BEX_{I,t}^{D}$ to see whether the downside volatility measure can serve as a qualified fear gauge or not.

We run the above regressions for each of 49 industry portfolios and report the arithmetic average of regression results in Table 5. The covariance matrix is computed according to Newey and West [33] to correct for any potential serial correlations in the error terms. On average, for both $\Delta SVX_{I,t}^{D}$ and $\Delta BEX_{I,t}^{D}$, all *β*_*1*_ are significantly less than 0 at 1% level, implying there is an inverse relation between the contemporaneous changes of volatility indices and those of portfolio returns. For both $\Delta SVX_{I,t}^{D}$ and $\Delta BEX_{I,t}^{D}$, *β*_*2*_ are also significantly less than 0 at 1% level. These results show that the response to different swings in portfolio returns is strongly asymmetric. This is consistent with findings reported above. By comparing the adjusted R-squared between Table 5 and Table 2, we can see that incorporating the downside risk into the volatility index can enhance monitoring effectiveness (37.9% and 37.6% for $\Delta SVX_{I,t}^{D}$ and $\Delta BEX_{I,t}^{D}$ in Table 5, and 36.7% for Δ*SVX*_*I*,*t*_ in Table 2). The results confirm that measures incorporating downside risk are better measures of fear gauge.

**Table 5:** Regression results of rate change of SVX^D^_I_ and ΔBEX_I_ against returns of industry portfolios.

	Dep Variable	Average of R2	Average of Intercept	Average of *β*_1_ for R_I,t_	Average of *β*_2_ for R_I,t_^-^
**Panel A**	**ΔSVX**^**D**^_**I**_	37.9%			
Coef			-0.001*	-0.514***	-0.222***
p-value			0.051	0.000	0.001
Std Err			0.000	0.019	0.031
**Panel B**	**ΔBEX**_**I**_	37.6%			
Coef			-0.001*	-0.397***	-0.171***
p-value			0.055	0.003	0.002
Std Err			0.000	0.015	0.025

This table presents the average OLS estimates of regressions in Eq (23). The regressions take the following general form

$\Delta SVX_{I,t}^{D}=\alpha +\beta_{1}R_{I,t}+\beta_{2}R_{I,t}^{-}+\varepsilon$

All coefficients, p-values, and standard errors are an average of the corresponding measures from 49 regressions on all industry portfolios. Independent variables include RI, daily return of the corresponding industry portfolio; and RI- daily return of the corresponding industry portfolio conditional on whether the return is below 0, i.e. R_I_ = min(R_I_, 0). The dependent variable is the daily return of SVXDI, where $\Delta SVX_{I,t}^{D}=\ln(SVX_{I,t}^{D}/SVX_{I,t}^{D})$. The return data are from 4 January 1996 to 29 April 2016. To correct for autocorrelation and heteroskedasiticity, we use the Newey-West estimator for covariance matric with automatically selected lags as in Newey and West [33].

***, **, and * denote significance at the 0.01, 0.05, and 0.10 level, respectively.

### Volatility forecasting: Out of sample evidence

Besides the in-sample forecasting evidence, here we further compare the volatility predictability by examining the out-of-sample tests for four volatility predictors: *HV*_*I*_, *VIX*_*I*_, *SVX*_*I*_, and *SVX*^*D*^_*I*_. Specifically, we use a rolling fixed window approach. Every day, each forecasting model is estimated with a fixed rolling window to obtain the one-month-ahead volatility forecast. To ensure robustness, we use a one-year window, a two-year window and a three-year window. The out-of-sample forecasting accuracy is judged by three criteria: root-mean-square error (RMSE), mean-absolute error (MAE) and mean-absolute-percentage error (MAPE).

Table 6 reports the estimation results for the average values of RMSE, MAE and MAPE across 49 industries in three different rolling windows. First, *HV*_*I*_ performs the worst among four volatility measures, regardless of criterion or rolling window. For instance, in the one-year rolling window case, the average RMSE values for *VIX*_*I*_, *SVX*_*I*_, and *SVX*^*D*^_*I*_ are all around 0.083, while it is 0.100 for *HV*_*I*_. Second, in almost all the scenarios, *SVX*^*D*^_*I*_ is the best predictor, with *SVX*_*I*_ and *VIX*_*I*_ being the second and third best predictors. Overall, the out-of-sample forecasting results reiterate the earlier in-sample test findings.

**Table 6:** Out-of-sample volatility forecasting results.

Panel A: 1-year rolling window			
	RMSE	MAE	MAPE
**HV**_**I**_	0.1005	0.0665	27.8261
**VIX**_**I**_	0.0838	0.0555	23.2998
**SVX**_**I**_	0.0832	0.0554	23.3153
**SVX**^**D**^_**I**_	0.0832	0.0554	23.3428
Panel B: 2-year rolling window			
	RMSE	MAE	MAPE
**HV**_**I**_	0.1086	0.0753	32.7627
**VIX**_**I**_	0.0879	0.0593	25.196
**SVX**_**I**_	0.0868	0.0587	24.9739
**SVX**^**D**^_**I**_	0.0867	0.0586	24.961
Panel C: 3-year rolling window			
	RMSE	MAE	MAPE
**HV**_**I**_	0.1102	0.0783	35.130
**VIX**_**I**_	0.0906	0.0621	26.763
**SVX**_**I**_	0.0894	0.0613	26.417
**SVX**^**D**^_**I**_	0.0891	0.0611	26.370

This table reports the out-of-sample forecasting results from January 1996 to April 2016 with a fixed rolling window approach. We report average values of RMSE, MAE and MAPE for 49 industries in the 1-year window, 2-year window, and 3-year window.

### Economic value of volatility timing

This section investigates the economic value of using various predictors to forecast monthly industry volatility. We start from assuming an investor has a negative exponential utility function:

$$U=-e^{-\gamma \tilde{w}},$$ (24)

where $\tilde{w}$ represents the wealth of the investor, and *γ* refers to the coefficient of the investor’s risk aversion. The expected utility takes the form of:

$$E(U)=\int_{-\infty }^{+\infty }-f(\tilde{w})e^{-\gamma \tilde{w}}d\tilde{w},$$ (25)

where $f(\tilde{w})$ is the density function of $\tilde{w}$, and expression of $f(\tilde{w})$ depends on the distribution of $\tilde{w}$.

In this study, for the sake of comparability with the literature on volatility timing [39–41], we choose normality for the distribution of $\tilde{w}$. Following [42] and [43], suppose that $\tilde{w}$ is distributed normally with mean, *μ*, and standard deviation, *σ*, the density of $\tilde{w}$:

$$f(\tilde{w})=\frac{e^{-\frac{{\left(\tilde{w}-\mu \right)}^{2}}{2\sigma^{2}}}}{\sigma \sqrt{2\pi }},$$ (26)

Substituting Eq 26 into Eq 25, and making a few rearrangements, we have:

$$E(U)=-e^{-\gamma (\mu -0.5\gamma \sigma^{2})},$$ (27)

Henceforth, the objective of the investor is to maximize the expression of *μ*−0.5*γσ*^2^, which leads to the mean-variance utility function.

Since in our setting, the investor’s wealth depends on the return of the portfolio invested, that is $\tilde{w}=w_{0}(1+R_{p})$, where *w*_0_ is the initial wealth, the maximum problem is equivalent to:

$${U[E}_{t}(R_{p,t+1}),\sigma_{p,t+1}^{2}]=E_{t}(R_{p,t+1})-0.5\gamma \sigma_{p,t+1}^{2},$$ (28)

where *E*_*t*_(*R*_*p*,*t*+1_) and $\sigma_{p,t+1}^{2}$ respectively are the conditional mean and variance of the portfolio returns. We set *γ* to a realistic estimate of 3, as suggested by [44] and [45]. We also use values of *γ* of 4 and 5 for robustness and sensitivity analysis.

In the volatility timing strategy, the investor allocates wealth between an industry portfolio and a risk-free asset using a volatility predictor to maximize utility gains. The portfolio return is *E*_*t*_(*R*_*p*,*t*+1_) = *r*_*f*,*t*+1_+*k*_*t*_(*E*_*t*_(*R*_*I*,*t*+1_)−*r*_*f*,*t*+1_), where *k*_*t*_ is the portfolio weight of industry portfolio *I*, *E*_*t*_(*R*_*I*,*t*+1_) is the conditional expected return of the industry portfolio, and *r*_*f*,*t*+1_ denotes the risk-free rate, which is known *ex-ante*. The portfolio variance is $\sigma_{p,t+1}^{2}=k_{t}^{2}\sigma_{I,t+1}^{2}$, where $\sigma_{I,t+1}^{2}$ denotes the conditional variance of industry portfolio *I*. The optimal weight for industry *I* is given by:

$$k_{t}=\frac{E_{t}(R_{I,t+1})-r_{f,t+1}}{\gamma \sigma_{I,t+1}^{2}},$$ (29)

The current study focuses on monthly realized volatility forecasting, so we assume the portfolio is rebalanced monthly. We set the month *t* expected return as the historical mean using return data up to period *t*. The expected variance of portfolio *I*, $\sigma_{I,t+1}^{2}$, is based on the two-year rolling out-of-sample forecast using Eq 10, with one of four different volatility measures (*HV*_*I*_, *VIX*_*I*_, *SVX*_*I*_, and *SVX*^*D*^_*I*_) as the predictor. The accuracy of volatility forecasting determines the performance of this volatility timing strategy. Our benchmark strategy is the buy-and-hold strategy of the respective industry portfolios, *I*.

To be consistent with our utility specification in Eq 24, we compare the performance of different strategies based on the certainty equivalent return (CER) gain of a volatility timing strategy relative to that of a naïve buy-and-hold strategy:

$$\mathrm{CER}=\left(R_{p}-0.5\gamma \sigma_{p}^{2}\right)-\left(R_{\mathrm{naive}}-0.5\gamma \sigma_{\mathrm{naive}}^{2}\right).$$ (30)

Intuitively, the CER gains of Eq 30 are the incremental management fees that the investor is willing to pay to invest in the volatility timing strategies based on the volatility forecasts over the buy-and-hold strategy.

Table 7 presents the average performance of 49 industries from January 1998 to April 2016. In Panel A, we assume there is no transaction cost. We first examine the basic case of *γ* equal to 3. The results on CER gains reveal that all the volatility timing strategies outperform the buy-and-hold strategy. $SVX_{I}^{D}$ again performs best: the investor is prepared to pay a hefty incremental annual management fee of 311 basis points bps to have access to predictive regression based on $SVX_{I}^{D}$, instead of the buy-and-hold strategy. In contrast, the investor is only willing to pay 249 bps for the strategy using *HV*_*I*._ Moreover, the management fees that the investor is willing to pay to be involved with the volatility timing strategy using $SVX_{I}^{D}$ increase from 331 bps (*γ* = 3) to 590 bps (*γ* = 4) and 866 bps (*γ* = 5). This result suggests that volatility timing is especially important for risk-averse investors.

**Table 7:** Out-of-sample portfolio performance: Monthly rebalancing.

Panel A: Without Transaction Cost			
	*γ* = 3	*γ* = 4	*γ* = 5
Predictor	CER (bps)	CER (bps)	CER (bps)
**BH**	**N/A**	**N/A**	**N/A**
**HV**_**I**_	249	528	816
**VIX**_**I**_	326	586	863
**SVX**_**I**_	330	589	865
**SVX**^**D**^_**I**_	331	590	866
Panel B: With Transaction Cost (0.25%)			
Predictor	CER (bps)	CER (bps)	CER (bps)
**BH**	**N/A**	**N/A**	**N/A**
**HV**_**I**_	193	486	816
**VIX**_**I**_	266	540	863
**SVX**_**I**_	269	543	865
**SVX**^**D**^_**I**_	270	544	866

This table reports the monthly out-of-sample portfolio allocation results from January 1998 to April 2016. We compare five strategies: buy-and-hold (BH), volatility timing based on HV_I_, volatility timing based on VIX_I_, volatility timing based on SVX_I_, and volatility timing based on SVX^D^_I_. We report average annualized certainty equivalent return (CER) gains for 49 industries under risk aversion coefficients of 3, 4, and 5. The CER gain (expressed in annualized basis points) is for a mean-variance investor who allocates between the industry portfolio and risk-free asset using the volatility timing strategy, relative to the naïve buy-and-hold passive strategy (BH). Panel A presents the results without transaction cost and Panel B presents the results counting for transaction cost.

The volatility timing strategy requires monthly rebalancing, so its performance might be sensitive to transaction costs. With this in mind, we analyze the impact of transaction costs on our results. Following [46] and [47], we define the transaction cost adjusted portfolio return as follows:

$${\bar{R}}_{p,t+1}=R_{p,t+1}-\rho (1+R_{p,t+1})|\Delta w_{t+1}|,$$ (31)

where ${\bar{R}}_{p,t+1}$ is the transaction cost adjusted portfolio return, *R*_*p*,*t*+1_ is the pre-adjusted portfolio return, *ρ* is the transaction cost parameter, and is set to be 0.0025, and Δ*w*_*t*+1_ is the change of weight from month *t* to month *t*+1.

Panel B of Table 7 presents the results for transaction cost adjusted performance. It clearly shows that, even when we account for transaction cost, volatility timing strategies based on *VIX*_*I*_, *SVX*_*I*_, and $SVX_{I}^{D}$, still largely outperform the buy-and-hold strategy and the volatility timing strategy based on *HV*_*I*._ Consistent with Panel A, $SVX_{I}^{D}$ generates the highest CER gain (269 bps) for *γ* = 3, and again, economic values are larger when the investor is more risk-averse (CER gains of 544 bps for *γ* = 4 and 829 bps for *γ* = 5).

In summary, this section uses a volatility timing strategy to show that the strong forecasting abilities of the industry volatility indices (*VIX*_*I*_, *SVX*_*I*_, and $SVX_{I}^{D}$) have significant economic value for investors.

## Conclusion

This paper is novel in that it proposes a general equilibrium framework to price volatility in the same manner as is the case for all securities in the market, following Arrow and Debreu [6]. Using state prices estimated from S&P 500 index options, we illustrate how we can derive *ex-ante* volatility measures *SVX*_*I*_ for industry portfolios, in which there are no traded options. The *SVX*_*I*_ measures generate superior forecasting abilities for the future realized volatility and serve as qualified fear gauges. We show that our approach is flexible and general by extending it to downside risk and upside opportunity. Finally, we demonstrate that the superior forecasting ability of our general equilibrium volatility measure can create significant economic value through a simple volatility timing strategy. Our findings, together with the fact that the industry volatility indices can be easily constructed under the general equilibrium framework, offer practitioners an appealing alternative tool for managing volatility. Our general equilibrium framework is not limited to pricing volatility, but can be applied to price any moments of the return distribution.

## Supporting information

S1 Data Data. (ZIP)

S1 Appendix Estimation of state prices. (DOCX)

S2 Appendix Equilibrium price, CAPM and Co-skewness pricing. (DOCX)

S3 Appendix Construction method of industry volatility. (DOCX)
