# Supplementary material for: A general equilibrium approach to pricing volatility risk
Source: PLoS One. 2019 Apr 12;14(4):e0215032. doi: 10.1371/journal.pone.0215032 (PMC6461293; doi:10.1371/journal.pone.0215032)
Supplement: S1 Appendix — (DOCX) [file pone.0215032.s002.docx]

**S1 Appendix: Estimation of state prices**

Breeden and Litzenberger [1] and Banz and Miller [2] show that state prices can be modelled as the second derivative of a European call option. This result comes from constructing a butterfly spread with a unit payoff. Assume that an investor longs one call option at a strike price of , one call option at , and shorts two calls at . The maturity of these calls is *T*. If the market price of the underlying asset is , the payoff of this portfolio would be and zero otherwise. Henceforth, when dividing this portfolio by , we can obtain a portfolio that produces a payment of $1 if the market price is , and zero otherwise.

The price of this portfolio is$P(M,T;\Delta M)=\frac{[c(M+\Delta M)-c(M)]-[c(M)-c(M-\Delta M)]}{\Delta M}$. If we divide the price $P(M,T;\Delta M)$ by $\Delta M$ and take a limit of $\Delta M$to zero, we can have:

(A1.1)

There are two different approaches to estimate Eq A1.1: (i) the Black and Scholes [3] risk-neutral framework, and (ii) the model-free approach using traded option prices. As suggested by Breeden and Litzenberger [1], the market portfolio is sufficient to represent different states in the economy. In this paper, we use the S&P 500 index (SPX) as the market portfolio.

1. The Black and Scholes [3] approach

Under the assumptions of Black and Scholes [3], the second derivative of a European call option equals to:

$\left.\frac{\partial^{2}C}{\partial K^{2}}\right|_{\text{K}=F_{s}}=\frac{e^{-rT}\varphi\left(d_{2}\right)}{K\sigma\sqrt{T}} ,$ (A1.2)

where$d_{2}=\frac{\ln\left((F_{0}-PVD)/K\right)-0.5\sigma^{2}T}{\sigma\sqrt{T}}$ is the familiar term in Black and Scholes [3] pricing formula; *ϕ(.)* refers to the standard normal probability density function evaluated at *d*2; *T* is the maturity of the option, which is set to be 30/365 in the current study; *r* and *σ* refer to the annualized risk-free rate and volatility of the SPX options; *σ* is estimated as the average of the implied volatilities of two at-the-money calls and two at-the-money put options, each with a maturity nearest to the 30-day period [4];*PVD* is the present value of the SPX dividends; and *K* is the strike price of the SPX options (i.e., the level of the SPX at which the state price is required).

The value of the contingent claim which generates the unit payoff if the price of the underlying asset is greater than or equal to some level $F_{s}$ can be calculated as follows*:*

$G\left( F_{s} \right)=\int_{F_{S}}^{\infty} \frac{e^{-rT}n\left( d_{2} \right)}{K\sigma\sqrt{T}}dK=e^{-rT}N\left[ d_{2}\left( K=F_{S} \right) \right]$ (A1.3)

We can then define the state price as the cost of a security with a unit payoff if the level of the underlying asset is between levels *Fs­* and *F­­s­+1*, using the Eq. A1.4 below:

$\phi\left( F_{s},F_{s+1} \right)=e^{-rT}\left\{ N\left[ d_{2}^{Black}\left( K=F_{s} \right) \right]-N\left[ d_{2}^{Black}\left( K=F_{s+1} \right) \right] \right\}$ (A1.4)

where SPX index level $F_{s+1}> F_{s}.$

In the calculation, the maximum and minimum values of state Y are bounded between 0.1 and 9,999. A state of 2,000 indicates the SPX will be at 2,000 after one month. State prices are assessed in increments of 0.1 between $F_{s}$ and $F_{s+1}$, representing 0.1 index point on the SPX.

1. The model-free approach using traded option prices

State prices can also be estimated using the numerical derivative from traded options as:

$\frac{\partial^{2}c(X,T)}{\partial X^{2}}|_{X=M}\approx\frac{c(M+\Delta M)-2c(M)-c(M-\Delta M)}{(\Delta M)^{2}}$ (A1.5)

Eq. A1.5 can be directly estimated using observed SPX option prices. However, Liu and O'Neill [5] point out several concerns regarding this approach, including generating zero state prices due to same deep out of the money (OTM) option prices, and negative state prices due to irrational bids for deep OTM options.

We adopt the simple Black and Scholes [3] analytical approach as it is simpler to use and less subject to numerical estimation issues. Liu and O'Neill [5] also show that the state prices under this approach lead to pricing results which are almost perfectly correlated with the model-free approach.

References:

1. Breeden DT, Litzenberger RH. Prices of state-contingent claims implicit in option prices. Journal of Business. 1978;51:621-51.

2. Banz RW, Miller MH. Prices for state-contingent claims: Some estimates and applications. Journal of Business. 1978;51:653-72.

3. Black F, Scholes M. The pricing of options and corporate liabilities. Journal of Political Economy. 1973;81(3):637-54.

4. Yan S. Jump risk, stock returns, and slope of implied volatility smile. Journal of Financial Economics. 2011;99(1):216-33.

5. Liu ZF, O'Neill MJ. State-preference pricing and volatility indices. Accounting & Finance. 2017;57(3):815-36.
