# Supplementary material for: A general equilibrium approach to pricing volatility risk
Source: PLoS One. 2019 Apr 12;14(4):e0215032. doi: 10.1371/journal.pone.0215032 (PMC6461293; doi:10.1371/journal.pone.0215032)
Supplement: S2 Appendix — (DOCX) [file pone.0215032.s003.docx]

**S2 Appendix: Equilibrium price, CAPM, and Co-Skewness**

The Arrow and Debreu [1] approach states that the value of the market is:

$P_{m}=\sum_{s=1}^{S} \varphi_{ms}F_{ms}$ (A2.1)

where $F_{tms}$ is the payoff on the market in state $s$ and $P_{m}$ is the current value of the market.

Using the set of state prices on the market $\varphi_{ms}$, we can now find the value of any asset. We simply find the expected value of asset $i$ conditional on the level of the market $E(F_{i}|F_{m})$, and multiply by the market state prices to obtain:

$P_{i}=\sum_{s=1}^{S} \varphi_{ms}E[F_{is}|F_{ms}]$ (A2.2)

The common way to project this expectation is the linear projection of$E(F_{i}|F_{m})=\alpha_{i}+\beta_{i}F_{m}$, which yields:

$P_{i}=\sum_{s=1}^{S} \varphi_{ms}[\alpha_{i}+\beta{}_{i}F_{ms}]$ (A2.3)

Now Eq. A2.3 looks very familiar and it closely resembles the CAPM in payoffs. However, it is different from CAPM because:

1. The state prices $\varphi_{ms}$ depend on the volatility of the market, the level of the market, the risk-free rate, and the usual determinants of option value. Therefore, the state prices could change continuously yet the CAPM assumes constant coefficients.

2. The conditional value of asset $i$, $E(F_{i}|F_{m})$ (Eq. A2.3) may not be linear in $F_{m}$. There are all sorts of non-linear ways of taking this conditional expectation (e.g., the excellent test of Friedman *et al.* [2]), but the CAPM only implies a linear projection with constant intercept and slope coefficients.

A nonlinear projection of the conditional expectation leads to the mean-variance-skewness model of Kraus and Litzenberger [3]:

(A2.4)

$P_{i}=\alpha_{rf}+\beta{}_{i}P_{m}+\gamma_{i}S_{m}$ (A2.5)

where $\beta{}_{i}$is the covariance of asset *i* with the market, $P_{m}$is the price of market risk, $\gamma_{i}$is the co-skewness of asset *i* with the market and $S_{m}$is the price of market co-skewness.

Reference

1. Arrow KJ, Debreu G. Existence of an equilibrium for a competitive economy. Econometrica. 1954;22:265-90.

2. Friedman J, Hastie T, Tibshirani R. The elements of statistical learning: Springer series in statistics Springer, Berlin; 2001.

3. Kraus A, Litzenberger RH. Skewness preference and the valuation of risk assets. The Journal of Finance. 1976;31(4):1085-100.
