# Supplementary material for: A general equilibrium approach to pricing volatility risk
Source: PLoS One. 2019 Apr 12;14(4):e0215032. doi: 10.1371/journal.pone.0215032 (PMC6461293; doi:10.1371/journal.pone.0215032)
Supplement: S3 Appendix — (DOCX) [file pone.0215032.s004.docx]

**S3 Appendix: Construction method of industry volatility**

We begin our construction by estimating a state-price volatility index for the whole market. This process is similar to the methodology described in Liu and O'Neill [1]. As the first step, we estimate the price of a security that pays off a dollar amount of $\log\left(\frac{M_{s}}{M_{0}}\right)^{2}$if the S&P 500 index moves to state $M_{s}$ from an initial value of $M_{0}$ in 30 days’ time. When summing across all possible future states, we arrive at the price of this asset in the following form:

(A3.1)

where $T$ is set to be exactly 30 calendar days (or equivalently, 22 trading days). ${SVX}_{M}^{2}$ can be viewed as a financial asset that pays off a dollar amount that is equivalent to the fair value of future variance. The factor 365/30 is to obtain an annualized volatility figure.

The second step involves estimating the relationship between each industry portfolio and the market portfolio, using a linear projection on the market model. That is, we are interested in the expected payoff for the industry conditional on the level of the index. The industry payoff here is the expected squared log returns$E\left[R_{I}^{2}|R_{M}^{2}\right]$. There are many ways to estimate this relationship [see e.g.,2]. Linear projection is the most common method in the extant literature [3], so we adopt this approach and use a simple linear least squares regression of daily squared industry returns on the squared S&P 500 market returns. In this setting, we use the market state prices and assume complete markets. The method captures the systematic components of industry volatility to the extent that the coefficient is allowed to vary over time. Specifically, we are interested in the alphas and betas in the following regression:

$R_{I}^{2}=\alpha_{I}+\beta_{I}R_{M}^{2}+\varepsilon_{I}$(A3.2)

The return $R_{I}$ is computed using the close value at the end of day. We estimate each beta using a two-year fixed rolling window. That is, on 505th day, we use the past two years (504 trading days) of return squared to estimate the beta in the above regressions.

The third step is to work out the individual industry portfolio volatility index based on the estimated alphas and betas and their corresponding volatility asset. To illustrate this, we substitute Eq. A3.2 into Eq. A3.1:

$SVX_{I}=\sqrt{\alpha_{I}\frac{365}{30}\sum_{s=1}^{S} \varphi_{ms}+\beta_{I}SVX_{M}^{2}}$ (A3.3)

The last step is to create an ad-hoc industry volatility index using the widely available CBOE volatility index *VIX*. *VIX* is a sum of weighted-average out-of-the-money S&P 500 put and call options. For a detailed discussion on *VIX*, we refer to Whaley [4]. As we do not have traded options for the industry portfolios, we cannot replicate the CBOE VIX methodology to reproduce the industry measures. Our ad-hoc estimation takes the following form by replacing$SVX_{M}^{2}$ with $VIX_{M}^{2}$:

$VIX_{I}=\sqrt{\alpha_{I}\frac{365}{30}\sum_{s=1}^{S} \varphi_{ms}+\beta_{I}VIX_{M}^{2}}$ (A3.4)

where $VIX_{M}$is the CBOE *VIX*.

Reference

1. Liu ZF, O'Neill MJ. State-preference pricing and volatility indices. Accounting & Finance. 2017;57(3):815-36.

2. Friedman J, Hastie T, Tibshirani R. The elements of statistical learning: Springer series in statistics Springer, Berlin; 2001.

3. Smith T, Walsh K. Why the CAPM is Half-Right and Everything Else is Wrong. Abacus. 2013;49(S1):73-8.

4. Whaley RE. Understanding the VIX. The Journal of Portfolio Management. 2009;35(3):98-105.
